# Antioxidative and Photo-Induced Effects of Different Types of N-Doped Graphene Quantum Dots

**DOI:** 10.3390/ma15196525

**Published:** 2022-09-20

**Authors:** Svetlana Jovanovic, Aurelio Bonasera, Sladjana Dorontic, Danica Zmejkoski, Dusan Milivojevic, Tamara Janakiev, Biljana Todorovic Markovic

**Affiliations:** 1 Institute of Nuclear Sciences—National Institute of the Republic of Serbia, University of Belgrade, Mike Petrovica Alasa 12-14, 11000 Belgrade, Serbia; 2 Department of Physics and Chemistry, Emilio Segrè, University of Palermo, 90128 Palermo, Italy; 3 Consorzio Interuniversitario Nazionale per la Scienza e Tecnologia dei Materiali (INSTM), Palermo Research Unit, Viale delle Scienze, bldg. 17, 90128 Palermo, Italy; 4 Faculty of Biology, University of Belgrade, Studentski Trg 16, 11158 Belgrade, Serbia

**Keywords:** graphene quantum dots, N-doping, gamma-irradiation, photoluminescence, photodynamic therapy, antioxidant, antibacterial effects

## Abstract

Due to the increasing number of bacterial infections and the development of resistivity toward antibiotics, new materials and approaches for treatments must be urgently developed. The production of new materials should be ecologically friendly considering overall pollution with chemicals and economically acceptable and accessible to the wide population. Thus, the possibility of using biocompatible graphene quantum dots (GQDs) as an agent in photodynamic therapy was studied. First, dots were obtained using electrochemical cutting of graphite. In only one synthetic step using gamma irradiation, GQDs were doped with N atoms without any reagent. Obtained dots showed blue photoluminescence, with a diameter of 19–89 nm and optical band gap of 3.23–4.73 eV, featuring oxygen-containing, amino, and amide functional groups. Dots showed antioxidative activity; they quenched •OH at a concentration of 10 μg·mL^−1^, scavenged DPPH• radicals even at 5 μg·mL^−1^, and caused discoloration of KMnO_4_ at 30 μg·mL^−1^. Under light irradiation, dots were able to produce singlet oxygen, which remained stable for 10 min. Photoinduced effects by GQDs were studied on several bacterial strains (*Listeria monocytogenes*, *Bacillus cereus*, clinical strains of *Streptococcus mutans*, *S. pyogenes*, and *S. sangunis*, *Pseudomonas aeruginosa*, and one yeast strain *Candida albicans*) but antibacterial effects were not noticed.

## 1. Introduction

Graphene quantum dots (GQDs) belong to the family of zero-dimensional materials due to the fact that their size is lower than 100 nm in all three directions: length, width, and height [1,2]. As a consequence, the localization of electrons is confined in all directions and becomes quantified. The GQD structure is the most similar to very-small-sized graphene oxide, with graphene in the core and oxygen-containing functional groups covalently attached to *sp*^2^-domains [3]. Functional groups in GQDs are usually carboxyl, carbonyl, hydroxyl, epoxy, and etoxy [4,5,6,7,8]. These groups are responsible for the high water solubility of GQDs, their biocompatibility, and their nontoxic behavior [9,10,11,12]. The graphene-like portion of the GQD structure is mainly responsible for its photoluminescence due to its semi-metallic behavior and nano size [13,14]. Particularly interesting and appealing is the strong PL emission of GQDs; the dots are resistive to photobleaching, show a longer PL emission compared to conventional bioimaging agents, and are chemically inert [13,14,15].

Good biocompatibility and emission in the visible part of the spectrum upon excitation make GQDs interesting for bioimaging applications [15,16,17,18]. Furthermore, other applications of GQDs in medicine are under the lens, e.g., as transport platforms for different drug molecules, for detection of biomolecules and pathogens [19], and as agents in photodynamic and photothermal therapy for the treatment of bacteria and cancer [20,21,22,23,24,25].

GQDs show unusual features; specifically, they are able to produce reactive oxygen species (ROS) when they are exposed to light by excitation and energy transfer to molecular oxygen [22,26]. Produced ROS is highly reactive and dangerous, leading to the death of cells. This is the fundamental concept of photodynamic therapy (PDT), an approach to the noninvasive treatment of both carcinoma and bacterial infection. There is a high demand for new agents for PDT that are water-soluble, biocompatible, nontoxic in the dark, highly efficient in ROS production during illumination, and able to be excited with longer wavelengths such as red and infrared [27,28].

Antioxidative properties have been noted for N-doped GQDs and highly graphitic GQDs [29]. An in vitro study confirmed that GQDs protect SH-SY5Y humane neuroblastoma cells from oxidative stress by reducing the intracellular level of •OH radicals, superoxide anion, and lipid peroxidation [30]. Other GQDs without any dopant atoms showed excellent antioxidative activity, as well as the ability to induce singlet oxygen formation when they were exposed to blue-violet light [31]. Our study proved that gamma irradiation increases the ability of GQDs to photogenerate singlet oxygen [32].

In this paper, we studied both the pro-oxidative and the antioxidative properties of several N-doped GQDs produced by gamma irradiation in different media. According to our earlier findings, which suggested that gamma irradiation causes chemical reduction and improves the ability of GQDs to produce ROS, we selected different reductive media for gamma irradiation. The ability of these dots to generate and quench ROS was studied.

## 2. Materials and Methods

### 2.1. Chemicals and Materials

Graphite electrodes (⌀ = 3.05 mm, and with 99.999% purity) were obtained from Ringsdorff-Werke GmbH (Bonn, Germany). Ethanol (96 vol%), sodium hydroxide, isopropyl alcohol (IPA), and acetone were purchased from Fisher Scientific (Loughborough, Leicestershire, UK). Ethylenediamine (EDA) (≥99.5%) was obtained from Carl Roth GmbH (Karlsruhe, Germany). Dialysis bags (MCWO 3.5 kDa) were supplied from Spectrum Laboratory Inc. (San Pedro St., Gardena, CA, USA).

### 2.2. Synthesis of Graphene Quantum Dots

GQDs were fabricated via the electrochemical oxidation of graphite electrodes, which were used as a starting carbon material [32]. This sample was denoted p-GQDs. Two types of N-doped GQDs were prepared by exposing p-GQDs to gamma rays at a dose of 25, 50, and 200 kGy in a mixture of water, IPA (1 vol.%), and EDA (4 vol.%), and these samples were named _25_GQD^IPA^-EDA, _50_GQD^IPA^-EDA, and _200_GQD^IPA^-EDA, respectively. After irradiation, samples were purified by dialysis until they reached pH 7.

The second set of samples was irradiated at 200 kGy in the presence of three different masses of EDA (1, 5, and 10 g). Then, water dispersion of p-GQDs in the concentration of 1 mg·mL^−1^ was sonicated for 30 min and exposed to gamma irradiation. Certain amounts of EDA were added. To remove oxygen, Ar was passed through the samples. These mixtures were irradiated at a dose of 200 kGy. As a radioactive source, a ^60^Co gamma source was used in the gamma sterilization facility at the Vinča Institute.

After irradiation, samples were dialyzed. Samples of GQDs irradiated at 200 kGy with different concentrations of EDA were labeled as _200_GQD-1EDA, _200_GQD-5EDA, and _200_GQD-10EDA.

### 2.3. Characterization of Graphene Quantum Dots

#### 2.3.1. Morphology Analysis of GQDs: Atomic Force Microscopy and Transmission Electron Microscopy

The surface morphology, topography, and size of GQDs were determined using atomic force microscopy (AFM). All water dispersions of GQDs at a concentration of 0.5 mg·mL^−1^ were used for deposition by spin-coating (3500 rpm, 1 min). Samples were placed on mica and recorded using Quesant AFM (Agoura Hills, CA, USA). The microscope was operated in the tapping mode, in air, and under room conditions. A typical silicon tip (NanoAndMore Gmbh, Wetzlar, Germany) was applied with a constant force of 40 N·m^−1^. Gwyddion 2.58 software was used for image analysis [33]. The accuracy of collected diameter distribution data was enhanced by the tip deconvolution procedure. This method is based on recalculation of GQD diameter into the so-called real diameter by applying the following equation:

r_c_ = r (cos θ_0_ + (cos^2^ θ_0_ + (1 + sin θ_0_)(−1 + (tan θ_0_/cos θ_0_) + tan^2^ θ_0_))^1/2^),

where r_c_ is the AFM radius of a particle, r is the particle radius, and θ_0_ is the mean half angle of the tip [33]. The histogram of diameter distribution was calculated using three different AFM images with the size of 25 × 25 μm^2^ for each GQD sample. For analysis of GQD size, another microscopic technique was used—transmission electron microscopy (TEM) JEOL JEM-2100F (JEOL, Ltd., Tokyo, Japan). Samples were prepared by depositing the GQD dispersion in ethanol in a concentration of 2 mg·mL^−1^ and drop-casting on copper grids. For image analysis, ImageJ software was used.

#### 2.3.2. Structural Analyses: Fourier-Transform Infrared Spectroscopy (FTIR) and X-ray Photoelectron Spectroscopy (XPS)

Surface functional groups of N-GQDs were investigated using FTIR spectroscopy. To obtain FTIR spectra, samples were dried into a powder and mixed with KBr. Then, samples were condensed into pastilles. Spectra were obtained using an FTIR spectrometer (Thermo Nicolet iS20, Waltham, Massachusetts, MA, USA), in the range of 4000–400 cm^−1^ at 32 scans per spectrum. The spectral resolution was 4 cm^−1^.

The ULVAC-PHI PHI500 VersaProbe II scanning microprobe (ULVAC-PHI, Inc., Chigasaki, Japan) was used to collect X-ray photoelectron spectra (XPS). GQD samples in the form of a powder were placed on Al support, and measurements were collected using an Al Kα source (1486.6 eV). The spot size was 100 µm, and the power was 25 W, with an acceleration of 15 kV. The takeoff angle was 45°. GQD spectra were measured using a dual neutralization system (both e^−^ and Ar^+^).

#### 2.3.3. UV/Vis Spectroscopy

UV/Vis absorption spectra of p-GQDs, _25_GQD^IPA^-EDA, _50_GQD^IPA^-EDA, _200_GQD^IPA^-EDA, _200_GQD-5EDA, and _200_GQD-10EDA were acquired using a LLG-uniSPEC 2 Spectrophotometer (Lab Logistic Group, Meckenheim, Germany). All spectra were recorded at room temperature in the wavelength range of 200–800 nm. GQDs were dispersed in ultrapure water in the concentration of 0.25 mg·mL^−1^ for measurements.

To calculate the values of the optical bandgap, Eg, the Tauc equation was used.

αhυ = B(hυ − Eg)^n^,

where “hυ” is the energy of the photon, B is a proportionality constant, and n is an exponent which is equal to 1/2 for direct transitions [34].

For analysis of the antioxidative properties of GQD samples, 2,2-diphenyl-1-picrylhydrazyl (DPPH), Rhodamine B (RHB), and KMnO_4_ tests were used. The DPPH assay was conducted by mixing GQD samples with freshly produced methanol solutions of DPPH at concentrations ranging from 0 to 200 g/mL. After 1 h in the dark, UV/Vis spectra were recorded using an LLG-uniSPEC 2 Spectrophotometer (Lab Logistic Group, Meckenheim, Germany). Ascorbic acid (vitamin C, AA) was used as a standard reference. Using the following equation, the radical-scavenging activities (RSA) of GQD samples were assessed:

RSA (%) = (A_C_ − A_GQDs_)/A_c_ × 100,

where A_C_ is the intensity of absorption of the control (DPPH in methanol), and A_GQD_ is the intensity of the absorption band of the mixture of GQDs with DPPH, also in methanol. The method is based on the fact that DPPH is a stable radical with high-intensity absorption at 518 nm. In the presence of an antioxidant, the intensity of the absorption band at 518 nm is lowered proportionally to the antioxidant concentration, whereby DPPH changes color from violet to yellow. Measurements were replicated three times. The RSA of each GQDs sample was determined using the RHB assay. Mixtures of GQDs in different mass concentrations (10–800 μg·mL^−1^), RHB, and H_2_O_2_ were subjected to 360 nm UV light for 1 h. After the incubation, UV/Vis spectra were recorded using an LLG-uniSPEC 2 Spectrophotometer (Lab Logistic Group, Meckenheim, Germany). AA was used as a standard. RSA values of GQDs samples were calculated using the following formula:

RSA (%) = A_GQDs_/A_c_ × 100,

where A_GQDs_ is the value of absorption at 554 nm measured from GQDs–RHB–H_2_O_2_ mixture, and A_c_ is that of the GQDs–RHB solution. Due to the contribution of GQD absorbance at higher concentrations, this absorption was subtracted. All measurements were replicated three times.

The antioxidative ability of GQDs was also tested using the KMnO_4_ reduction assay. We used the previously reported protocol [29,35] and modified the concentration of KMnO_4_. We mixed an acidified solution of KMnO_4_ at pH 3 in the concentration of 300 mM and added different amounts of GQDs, from 2.5 to 1000 μg·mL^−1^. Mixtures were held in the dark for 1 h, and the UV/Vis spectra were recorded. When KMnO_4_ is reduced, it changes color from purple to colorless.

#### 2.3.4. Photoluminescence (PL) Spectroscopy

For PL measurement, N-GQDs samples were dispersed in the concentration of 0.04 mg·mL^−1^ in ultrapure water. Emission spectra of N-GQDs were taken at room temperature and atmospheric pressure using the HORIBA Jobin Yvon’s Fluoromax-4 spectrometer (Horiba, Kyoto, Japan) at different excitation wavelengths from 320 to 380 nm in the range of 340–580 nm. The excitation slit was 8 nm, while the emission slit was 2 nm. The integration time was 0.5 s.

#### 2.3.5. Electron Paramagnetic Resonance (EPR) Spectroscopy

The ability of dots to quench and produce reactive oxygen species (ROS) was monitored using electron paramagnetic resonance (EPR) spectroscopy. EPR Spectrometer MiniScope 300, Magnettech, Berlin, Germany, was operated at a nominal frequency of 9.5 GHz, the microwave power was 0.32 mW (microwave attenuation of 25 dB), and the modulation amplitude was 0.2 mT.

To investigate the potential of GQDs to scavenge hydroxyl radical (•OH), 5,5-dimethyl-1-pyrroline *N*-oxide (DMPO) and the Fenton reaction were used [31]. DPMO in EPR is inactive; however, with •OH radical, it forms the spin adduct DMPO/•OH, with a characteristic EPR signal. •OH radicals were produced in a classical Fenton reaction (Fe(II) and H_2_O_2_). The EPR spectra were recorded at 1 min after initiating the generation of •OH, by adding H_2_O_2_. The amount of •OH was quantitatively determined from the integrated intensity of EPR signals. To explore the ability of GQD to quench •OH radicals, to the DMPO/Fenton reaction solution, 0.2 wt.% dots were added to DMPO in 12.5 mM, 1.25 mM, and 0.146 mM concentrations.

Production of singlet oxygen (^1^O_2_) was measured using TEMP as a spin trap. GQDs powdered samples in 0.2 wt.% were mixed with 30 mM of TEMP ethanol solution. Then, samples were exposed to UV light, and EPR spectra were measured. In this way, formation of ^1^O_2_ was followed by a specific reaction between singlet oxygen and TEMP, wherein a stable radical adduct, TEMP-^1^O_2_ (or TEMPO), was produced. Mixtures of TEMP and GQDs were air-equilibrated before measurement. We obtained reference spectra of TEMP only and of TEMP-GQDs in dark. Then, samples were exposed to UV/Vis light (λ > 360 nm) for 15 min and spectra were recorded every 5 min. Signals in each EPR spectra of TEMPO produced in solutions of GQDs were analyzed by calculating their integrated intensity.

#### 2.3.6. Photo-Induced Antibacterial Activity

N-GQD was evaluated as an antibacterial drug against two Gram-positive reference strains (*Listeria monocytogenes* ATCC 13932 and *Bacillus cereus* ATCC 11778) and three clinical strains of *Streptococcus mutans*, *S. pyogenes*, and *S. sangunis*. The antimicrobial properties of these GQDs were also tested against Gram-negative reference strains *Pseudomonas aeruginosa* ATCC 10145 and one yeast strain *Candida albicans* ATCC 10231. Phosphate buffer (1× PBS, phosphate saline buffer) was used for the preparation of bacterial suspensions, where the final concentration of bacterial strains was 10^8^ CFU·mL^−1^.

The minimum inhibitory concentration (MIC) and the minimum bactericidal concentration (MBC) of the selected nanoparticles were determined using the microdilution method [36]. Bacterial suspensions were cultured in Luria–Bertani (LB) medium. The final volume of bacterial inocula was 200 µL. A sterility control was also prepared. GQDs in the concentration range of 0.8–0.025 mg·mL^−1^ were mixed with bacterial suspensions and transferred into the 96-well microtiter plates. Mixtures were incubated for 16 h at 37 °C and exposed to light irradiation (470 nm, 15 W). The distance from the light source was 20 cm. The light irradiance in the proximity of the sample was 19 mW·cm^−2^. In addition to the negative control, a sterility control was prepared. The optical density (OD) of irradiated samples was read on a spectrophotometer (Epoch microplate spectrophotometer, Agilent, Santa Clara, CA, USA). MIC and MBC were determined by adding 22 µL of resazurin to the microtiter plates, which were incubated for an additional 2 h at 37 °C. According to the resazurin reaction, the lowest concentration without a color change was defined as MIC. The lowest concentration that did not show bacterial growth after subculturing and incubation was defined as MBC.

## 3. Results and Discussion

**Figure 1:**
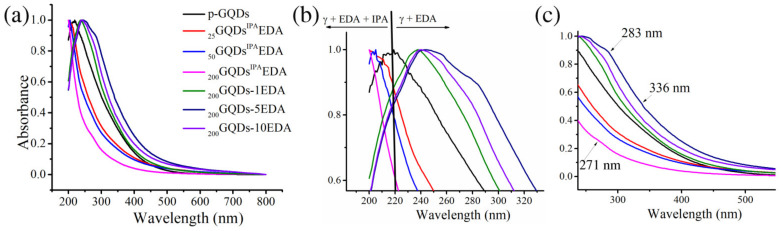
UV/Vis spectra of p-GQDs, _25_GQD^IPA^-EDA, _50_GQD^IPA^-EDA, _200_GQD^IPA^-EDA, _200_GQD-5EDA, and _200_GQD-10EDA (**a**); enlarged low-wavelength regions: 200–300 nm (**b**) and 250–500 nm (**c**).

The optical properties of N-doped GQDs were investigated using UV/Vis spectroscopy, and these results are presented in Figure 1. As can be seen in Figure 1a, all spectra had a similar shape, with high absorption in the UV part of the spectrum, decreasing at a higher wavelength. The center of the absorption maximum reflected the π → π * transition in *sp*^2^ domains of the graphene-like core [37], which was located at 220 nm for p-GQDs. The enlargement presented in Figure 1b clearly shows that the absorption peak was shifted to a higher wavelength when GQDs were functionalized under irradiation in presence of sole EDA (between 242 and 248 nm), while irradiation in the presence of both EDA and IPA caused a shift to lower wavelengths, with bands centered around 200 for _200_GQDs^IPA^-EDA, 204 for _50_GQDs^IPA^-EDA, and 207 nm for _25_GQDs^IPA^-EDA. The second shoulder band stemming from n → π * transitions of a carbonyl group [38,39] could not be observed in p-GQDs, while it was located around 271 nm for _200_GQDs^IPA^-EDA and the other two samples irradiated in the same media. For _200_GQDs-1EDA, this band could not be observed, while, in the case of _200_GQDs-5EDA and _200_GQDs-10EDA, this band was shifted to a higher wavelength, at 280 and 283 nm, respectively. So-called “n” electrons are two pairs of nonbonding electrons at the O-atom in C=O, and the n → π * transition occurs when one of these electrons is excited and hops to a higher-energy π * orbital [40]. Spectra show that GQD irradiated with EDA had higher adsorption, suggesting a larger number of surface oxygen states (C=O) compared to p-GQDs [41]. The theoretical study showed that, with an increase in the size of the π-conjugated system, GQDs were able to absorb light over longer wavelengths and caused a red shift in the absorption spectrum [42], while dopants disrupted π-conjugated domains and lowered the absorptions. In our study, irradiation with both IPA and EDA at all applied doses induced a decrease in the absorption, leading to the conclusion that π-domains in these dots were reduced. On the other hand, when dots were irradiated with different amounts of EDA, absorption in the UV part was improved, suggesting that, for these dots, π-domains were restored and increased.

**Figure 2:**
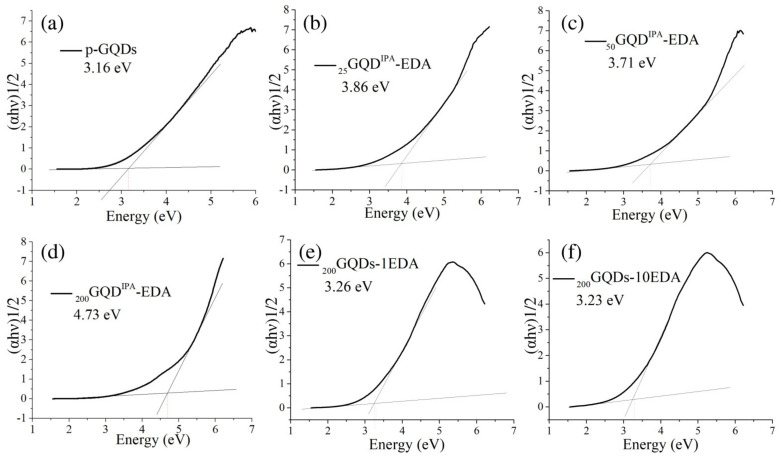
Tauc plots graphs for p-GQDs (**a**), _25_GQD^IPA^-EDA (**b**), _50_GQD^IPA^-EDA (**c**), _200_GQD^IPA^-EDA (**d**), _200_GQD-1EDA (**e**), and _200_GQD-10EDA (**f**).

Optical bandgaps (E_g_) of GQDs samples were calculated using Tauc plots derived from UV/Vis spectra, and these results are presented in Figure 2. For nonmodified dots, the bandgap was evaluated as 3.16 eV (Figure 2a), and, after irradiation at 200 kGy in the presence of EDA, there were no large changes in this parameter (Figure 2e,f). However, in the case of gamma irradiation with IPA and EDA, at all applied doses, the value of the optical bandgap was increased to 3.86, 3.71, and 4.74 for doses of 25, 50, and 200 kGy (Figure 2b–d), respectively. Obtained values are in agreement with data obtained from the literature [40,41,42]. We previously observed that gamma irradiation of GQDs with a water solution of IPA induced the increase in the value of the optical bandgap due to an increase in oxygen groups, as well as in the interlayer bonding between graphene sheets in the cores of GQDs [43]. The second reason for the increase in the E_g_ values could be due to the reduction in size of *sp*^2^-domains [44]. The analysis of UV/Vis absorption spectra indicated a decrease in *sp*^2^-domains for dots irradiated in the IPA-EDA mixture, which was also probably the reason for the increase in E_g_ values.

**Figure 3:**
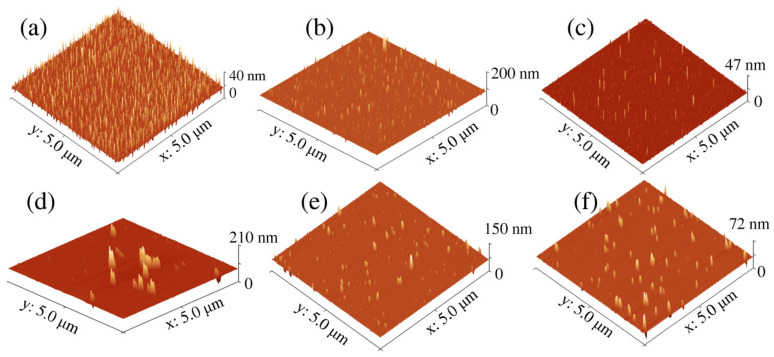
AFM images of p-GQDs (**a**), _25_GQD^IPA^-EDA (**b**), _50_GQD^IPA^-EDA (**c**), _200_GQD^IPA^-EDA (**d**), _200_GQD-5EDA (**e**), and _200_GQD-10EDA (**f**).

To study the morphology of GQDs, AFM images were obtained (Figure 3a–f). The p-GQDs were well dispersed, and small dots were noted. In the case of _25_GQD^IPA^-EDA and _50_GQD^IPA^-EDA (Figure 3b–c), a similar situation was observed. While most GQDs were small in diameter and well dispersed, agglomerates of large height (up to 200 nm) could be sporadically observed. In the case of _200_GQD^IPA^-EDA (Figure 3d), large agglomerates with a height of around 200 nm are more abundant. In the case of _200_GQD-5EDA (Figure 3e), small-size GQDs were most dominant, whereas in the case of _200_GQD-10EDA, larger agglomerates were more frequently detected.

In Table 1, the values of average lateral size and height for GQDs samples are presented. It can be observed that both height and diameter increased with gamma-irradiated dose.

**Figure 4:**
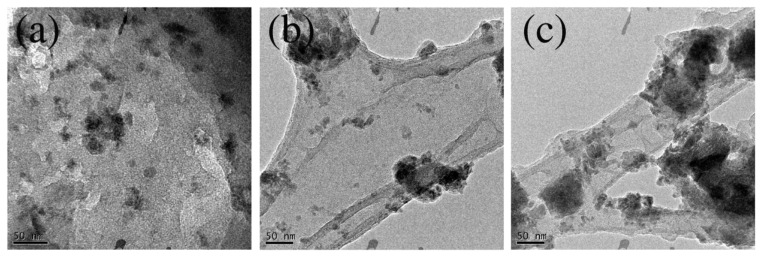
TEM images of _25_GQD^IPA^-EDA (**a**), _200_GQD-5EDA (**b**), and _200_GQD-10EDA (**c**).

In Figure 4, TEM micrographs of _25_GQD^IPA^-EDA (a), _200_GQD-5EDA (b), and _200_GQD-10EDA (c) are shown. Figure 4a reveals small particles with a size between 20 and 30 nm, while, in Figure 4b,c, larger particles and groups of particles can be observed. These results are in agreement with AFM analysis which showed that GQDs irradiated with IPA and EDA at low doses were small compared to those irradiated with EDA.

**Figure 5:**
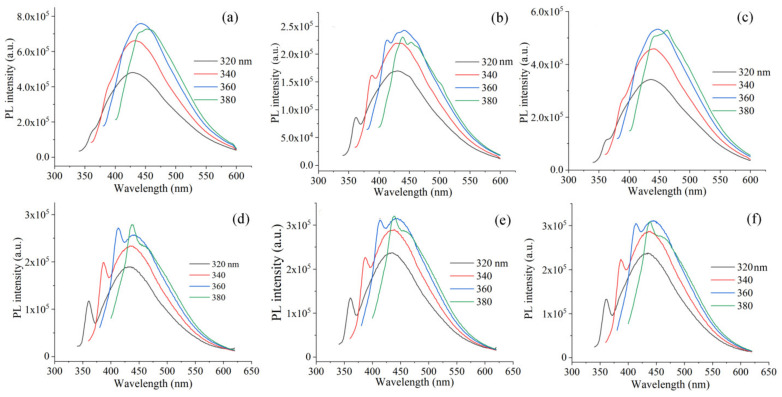
PL spectra of _25_GQD^IPA^-EDA (**a**), _50_GQD^IPA^-EDA (**b**), _200_GQD^IPA^-EDA (**c**), _200_GQD-1EDA (**d**), _200_GQD-5EDA (**e**), and _200_GQD-10EDA (**f**) recorded at excitation wavelengths of 320, 340, 360, and 380 nm.

In Figure 5, the PL spectra of modified dots are presented upon excitation from 320 to 380 nm. Samples showed a center of emission between 430 and 490 nm, which was excitation-dependent. This kind of behavior was described in earlier reports [43,44], and it was rationalized as the combined result of variations in size, *sp*^2^-domains, and level of functionalization in the samples. The highest intensity of PL emission bands was measured at an excitation wavelength of 360 nm; for sample _25_GQDs^IPA^-EDA, it was 759,083, whereas, it was 242,419 for _50_GQDs^IPA^-EDA and 532,427 for _200_GQDs^IPA^-EDA. In the case of samples irradiated at 200 kGy with EDA, similar intensities of the absorption bands were 256,274, 314,855, and 311,290 for _200_GQD-1EDA, _200_GQD-5EDA, and _200_GQD-10EDA, respectively. These results show that the highest intensity of PL emission was measured for sample _25_GQDs^IPA^-EDA. Thus, the selected condition regarding the dose and mixture of solvents as a medium for irradiation led to the largest improvement in PL intensity.

**Figure 6:**
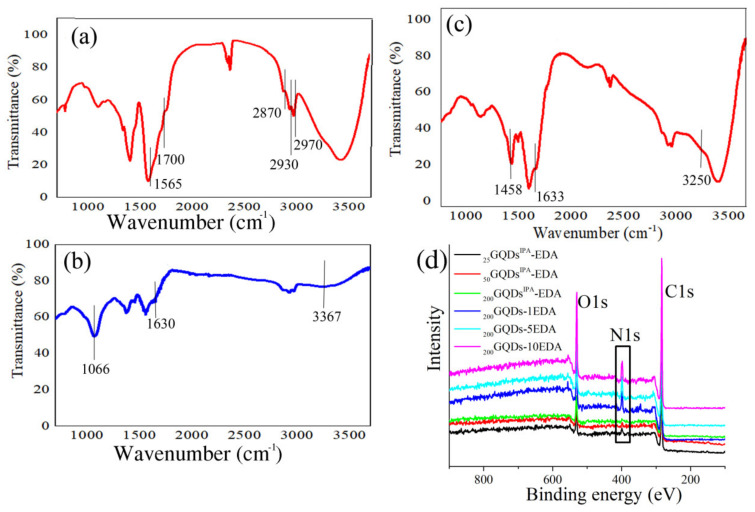
FTIR spectra of p-GQDs (**a**), _25_GQDs^IPA^-EDA (**b**), and _200_GQDs-10EDA (**c**) samples; XPS spectra of _25_GQDs^IPA^-EDA, _50_GQDs^IPA^-EDA, _200_GQDs^IPA^-EDA, _200_GQDs-1EDA, _200_GQDs-5EDA, and _200_GQDs-10EDA (**d**).

The structure of GQDs was investigated using FTIR spectroscopy (Figure 6a–c). In the FTIR spectrum of p-GQDs, the following bands were observed: 1565 cm^−1^ stretching vibrations of C=C bonds in aromatic domains; 2970, 2931, and 2870 cm^−1^ stretching vibrations from C–H bonds in –CH and –CH_2_ groups; 1700 cm^−1^ C=O bonds in carbonyl groups. In the case of _25_GQDs^IPA^-EDA, the band at 1700 cm^−1^ was shifted to 1630 cm^−1^ as a result of the reduction, while a new band was observed at 3360 cm^−1^, assigned to stretching vibrations of N–H bonds in amino groups; furthermore, new bands at 1066 cm^−1^ stemmed from C-N stretching vibrations. For sample _200_GQDs-10EDA, the band at 1633 cm^−1^ was ascribed to the stretching vibrations of C=O bonds in amide functional groups, the new band at 1458 cm^−1^ stemmed from C–N bending vibration in N–C=O functional groups, and the new band at 3250 cm^−1^ was attributed to stretching vibrations of N–H bonds in amino groups. FTIR analysis showed that GQDs irradiated with both IPA and EDA were partially reduced with amino groups in the structure, while, in the case of irradiation with EDA, amino and amide bonds were detected. These samples were analyzed using XPS, and the spectra are presented in Figure 6d; the fitted peaks confirmed the functional groups reported in the FTIR spectra of previous research articles from our group [38,45]. In the spectra of all gamma-irradiated GQDs, the presence of the elements carbon (C 1*s*) and oxygen (O 1*s*) at around 284 eV and 532 eV, respectively, could be observed. In the samples _25_GQDs^IPA^-EDA, _50_GQDs^IPA^-EDA, and _200_GQDs^IPA^-EDA, an additional, low-intensity peak in the region around 399 eV was observed, while this peak was present in the same region but with a higher intensity for samples _200_GQDs-1EDA, _200_GQDs-5EDA, and _200_GQDs-10EDA. This peak stemmed from N 1*s* and indicated the high atomic percentage of N in samples irradiated with EDA (~7%), whereas, in samples irradiated with EDA and IPA, only around 3% N was detected in the structure [38,45].

**Figure 7:**
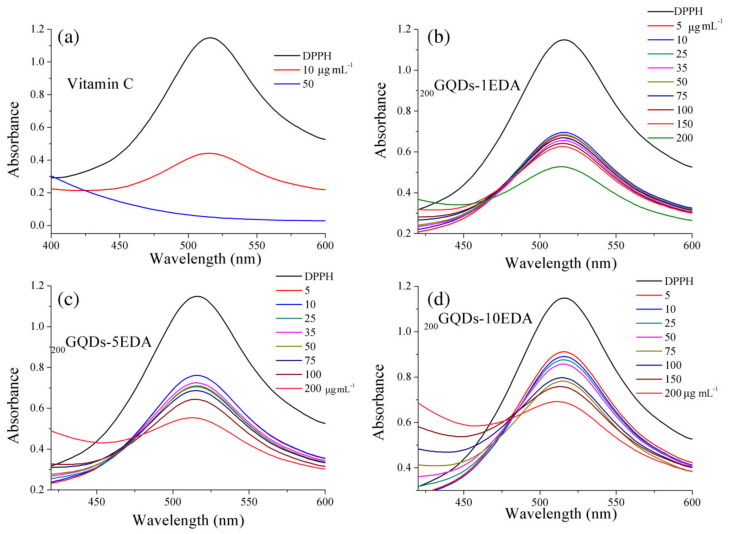
DPPH•-scavenging assay: UV/Vis absorption spectra of 100 μM DPPH• methanol solution with vitamin C at 10 and 50 μg·mL^−1^ (**a**), and with GQDs samples _200_GQDs-1EDA (**b**), _200_GQDs-5EDA (**c**), and _200_GQDs-10EDA (**d**), at concentration values ranging from 5 to 200 μg·mL^−1^ after 1 h in dark.

To investigate the reactivity and ability of modified dots to scavenge nitrogen-centered radical species, a DPPH test was conducted, and the results are presented in Figure 7. At can be seen, all samples of gamma-irradiated dots induced a decrease in the absorption at 517 nm, which is a characteristic of DPPH radicals. A decrease in the intensity of this band was a result of bond creation between the N-centered radical of DPPH and protons [46] from the GQDs samples, leading to discoloration of the molecule. UV/Vis spectra of mixtures of DPPH• and GQDs samples revealed that they had the ability to quench DPPH radicals. A large decrease in the band at 517 nm was noted, even at a concentration of 5 μg·mL^−1^, for all three samples of dots irradiated in EDA at 200 kGy. We previously studied the DPPH•-scavenging ability of p-GQDs, _25_GQD^IPA^-EDA, _50_GQD^IPA^-EDA, and _200_GQD^IPA^-EDA [38] and showed that all dots were potent DPPH• quenchers. Similar results were obtained for highly crystalline GQD through hydrothermal condensation of pyrene [29].

**Figure 8:**
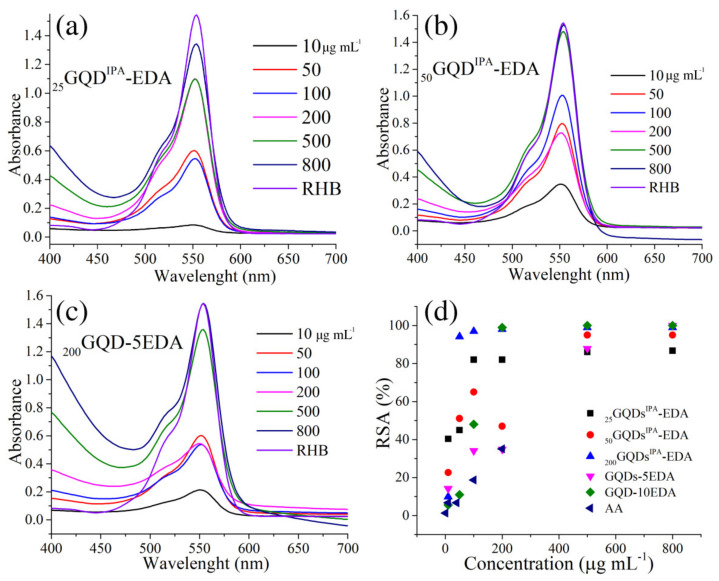
RHB test: _25_GQD^IPA^-EDA (**a**), _50_GQD^IPA^-EDA (**b**), _200_GQD-5EDA (**c**), and RSA as a function of GQDs concentration (**d**).

The ability of modified dots to quench •OH radicals was studied using RHB, and these results are presented in Figure 8. This assay is based on the discoloration of RHB under UV irradiation and hydrogen peroxide [47]. Photoinduced production of •OH radicals leads to bleaching of RHB. When an antioxidant is present in the system, it prevents RHB from losing its color by quenching the •OH radicals produced in the system. Each test tube contained the same concentrations of RHB and H_2_O_2_ (0.02 and 0.03 mM, respectively), while those of the dots varied, from 10 to 800 μg·mL^−1^. The •OH-scavenging activity of GQDs was previously reported [29,47,48]. In the mixture of RHB, H_2_O_2,_ and different concentrations of GQDs, after 1 h of UV irradiation, the intensity of the band at 554 nm increased with GQDs concentration (Figure 8b–c). For comparison, the same mixtures were prepared with ascorbic acid (Figure 8d, AA) as a standard antioxidant. With RHB, the spectrum obtained for the control solution without peroxide and irradiation was recorded. In the presence of 10 μg·mL^−1^ GQDs samples, almost total bleaching of RHB was observed, while the intensity of the band at 554 nm was increased with the increase in GQD concentration. This is an indication of the GQD protective effect against •OH radical oxidation of RHB. The largest changes were noticed at 50 μg·mL^−1^ concentration, whereby _200_GQDs^IPA^-EDA showed the highest intensity and •OH-scavenging activity, while other samples of GQD irradiated with both EDA and IPA showed a medium activity, and samples irradiated with EDA (Figure 8d) had a lower •OH-scavenging activity.

**Figure 9:**
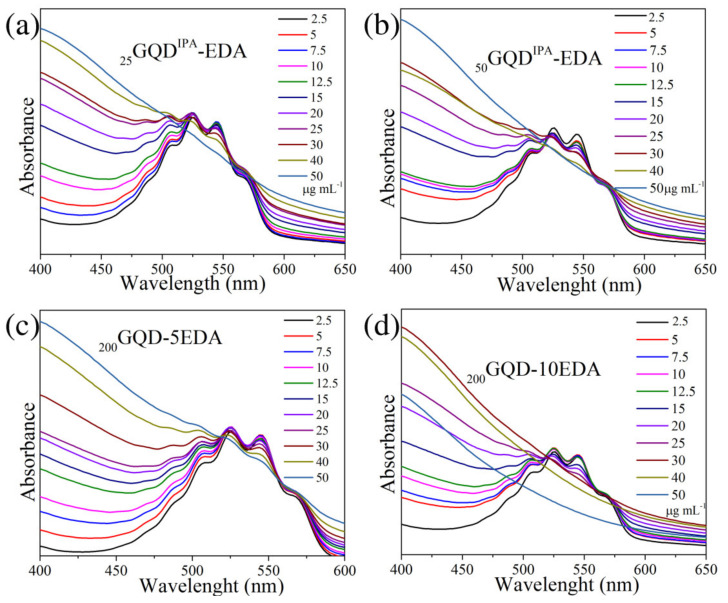
KMnO_4_ reduction test: UV/Vis spectra of mixtures of KMnO_4_ (300 mM) with GQDs samples _25_GQD^IPA^-EDA (**a**), _50_GQD^IPA^-EDA (**b**), _200_GQD-5EDA (**c**), and _200_GQD-10EDA (**d**) added in the concentrations indicated in the images, recorded after 1 h of incubation in the dark.

The ability of GQDs samples to act as antioxidants was studied using a KMnO_4_ reduction assay, and these results are presented in Figure 9. The color of the acidified solution of KMnO_4_ changes from purple to colorless in the presence of antioxidants due to the reduction of Mn^7+^ (purple) to Mn^2+^ ions, with a band at 440 nm [49,50]. UV/Vis absorption spectra of the 300 μM KMnO_4_ water solution with different concentrations of _25_GQD^IPA^-EDA, ranging from 2.5 to 50 μg·mL^−1^ (Figure 9a), show that, with the increase in GQDs concentration, the intensity of KMnO_4_ band decreased, while that of the band at the shorter wavelength increased. All GQDs samples showed the ability to bleach the KMnO_4_ solution, and the best results were obtained for sample _200_GQDs-10EDA, which was able to most efficiently induce the discoloration of KMnO_4_ solution even at a concentration of 30 μg·mL^−1^ (Figure 9d). It was previously observed that *sp*^2^-rich GQDs are even better antioxidants than ascorbic acids due to their ability to transfer electrons to KMnO_4_ [29]. The same study showed that GQDs with N-atoms incorporated in graphene sheets showed a mild oxide radical-scavenging activity, where those that were *sp^3^* C-rich did not demonstrate any activity, nor did graphene oxide [29,47]. The analysis of the optical bandgap indicated that dots irradiated in the IPA–EDA mixture had smaller *sp*^2^ domains, while the same analysis suggested that these domains were larger in dots irradiated in EDA. This is probably the reason why the samples _200_GQD-10EDA and _200_GQD-5EDA showed a better scavenging activity than GQDs irradiated in both EDA and IPA. Both types of dots contained electron-rich amino and amide functional groups, which were able to transfer the electrons to KMnO_4_ and act as antioxidants.

**Figure 10:**
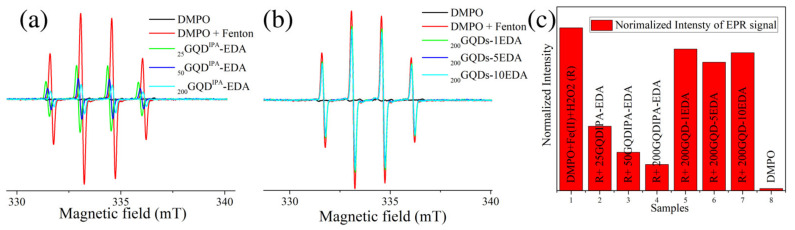
DMPO assay for OH-scavenging activity: EPR spectra of DMPO, Fe^2+^, and H_2_O_2_ mixed with _25_GQD^IPA^-EDA, _50_GQD^IPA^-EDA, and _200_GQD^IPA^-EDA (**a**), or _200_GQDs-1EDA, _200_GQDs-5EDA and _200_GQDs-10EDA (**b**); normalized intensities of DMPO–OH signals (**c**).

To investigate the ability of GQDs to scavenge •OH radials, we performed a test combining the Fenton reaction and EPR spin trap DMPO. Specifically, Fe^2+^ was mixed with H_2_O_2_, and OH radicals were quickly formed. Spin trap DMPO was added to measure produced OH radicals due to DMPO–OH radical adduct formation [47]. This product is EPR active and shows a characteristic signal, whereas DMPO does not show any signal (Figure 10a). When Fe^2+^ and H_2_O_2_ were added, DMPO–OH radical production was observed (red signal in Figure 10a). When OH radical quencher was present in this mixture, the signal from DMPO–OH radicals was expectedly reduced. When _25_GQD^IPA^-EDA, _50_GQD^IPA^-EDA, and _200_GQD^IPA^-EDA were added, each of the samples caused a decrease in the DMPO–OH radical intensity (Figure 10a). These results indicate that GQDs induced a decrease in OH radical production. On the other hand, samples _200_GQDs-1EDA, _200_GQDs-5EDA, and _200_GQDs-10EDA caused very small changes in the intensity of the DMPO–OH signal (Figure 10b). When all samples were compared (Figure 10c), it became clear that dots irradiated with EDA and IPA showed a high •OH scavenging activity, while dots irradiated with EDA at 200 kGy were not very efficient in OH radical scavenging.

**Figure 11:**
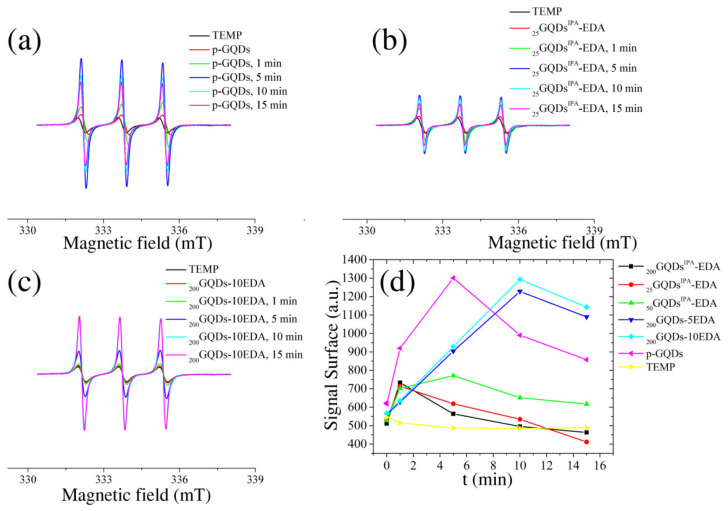
EPR spectra recorded with TEMP as a spin trap for p-GQDs (**a**), _25_GQD^IPA^-EDA (**b**), and _200_GQD-10EDA (**c**); the calculated peak surface of each signal as a function of time of illumination (**d**).

The photoinduced pro-oxidative activity of GQDs samples was studied using the EPR spin trap method. Specifically, time-accumulating EPR spectra were collected during irradiation of a solution of TEMP that contained 0.2 wt.% of gamma-irradiated GQDs. In Figure 11a, the EPR spectra of TEMP and TEMP mixed with non-irradiated GQDs are presented. It is clear that, with an increase in the time of UV light exposure, the signal characteristic for TEMPO increased proportionally. In the case of _25_GQDs^IPA^-EDA (Figure 11b), the increase in TEMPO signal with UV light exposure was evident but much lower compared to p-GQDs. For sample _200_GQDs-10EDA (Figure 11c), the production of TEMPO was larger than for _25_GQDs^IPA^-EDA and similar to p-GQDs. Figure 11d displays the values obtained by calculating the signal surface. Dots irradiated with IPA and EDA were unable to produce singlet oxygen under UV light, while dots irradiated with EDA at 200 kGy generated singlet oxygen. The production increased with time in the first 10 min before stagnating. A similar situation was previously observed [32]. The saturation of singlet oxygen production is probably due to the consumption of molecular oxygen from the medium. These results indicated that both _200_GQDs-5EDA and _200_GQDs-10EDA have the potential for application in photodynamic therapy as photosensitizers.

### Antimicrobial Activity

In the tested range of concentrations (0.8 to 0.025 mg·mL^−1^), _25_GQD^IPA^-EDA, _50_GQD^IPA^-EDA, _200_GQD-1EDA, and _200_GQD-10EDA samples did not show antimicrobial activity (Table 2).

In parallel, for _25_GQD^IPA^-EDA, _50_GQD^IPA^-EDA, and _200_GQD-1EDA, OD values were obtained to determine whether there was a reduction in OD values in the negative control (Table 3). In most cases, a higher optical density was observed in the treatment compared to the control. The increase in optical density was likely to change the nanoparticles; hence, this method was not considered reliable for assessing antimicrobial activity. Therefore, the results of antimicrobial activity were determined exclusively from the resazurin reaction and reinoculation into the appropriate nutrient medium.

## 4. Conclusions

Gamma irradiation of GQDs in the presence of EDA and a mixture of EDA and IPA showed very different properties; GQDs irradiated with EDA were able to quench DPPH radicals, very efficiently reduce KMnO_4_, and induce discoloration of GQDs dispersion, whereas dots irradiated with IPA and EDA were able to quench OH radicals. Particularly efficient OH quenching was observed for _200_GQDs^IPA^-EDA. The EPR study showed that the production of singlet oxygen was highest after _200_GQDs-5EDA and _200_GQDs-10EDA samples were illuminated for around 10 min, while samples _25_GQDs^IPA^-EDA, _25_GQDs^IPA^-EDA, and _25_GQDs^IPA^-EDA were unable to produce ROS under photoexcitation. The production of singlet oxygen increased for 10 min and then stagnated due to oxygen consumption from the medium. When bacterial strains were treated with GQDs and illuminated, toxic effects were not observed. It was assumed that the proposed conditions did excite GQDs. Considering the observed antioxidative capacity, GQDs might have the potential for application in the treatment of conditions related to oxidative stress.

## Figures and Tables

**Table 1:** The average diameter and the height of GQDs as calculated from AFM images.

Sample	Height (nm)	Diameter (nm)
_25_GQD^IPA^-EDA	0.86 ± 0.29	19.00 ± 4.91
_50_GQD^IPA^-EDA	1.08 ± 0.51	32.92 ± 6.70
_200_GQD^IPA^-EDA	9.3 ± 8.99	89.19 ± 24.80
_200_GQD-1EDA	1.32 ± 0.63	33.46 ± 7.22
_200_GQD-5EDA	2.49 ± 0.85	35.73 ± 8.85
_200_GQD-10EDA	1.65 ± 0.71	45.74 ± 7.86

**Table 2:** Minimum inhibitory concentrations of GQD-EDA/1 g and GQDs-EDA/10 g (in mg·mL^−1^).

Bacterial Strain	_25_GQD^IPA^-EDA		_50_GQD^IPA^-EDA		_200_GQD-1EDA		_200_GQD-10EDA	
	MIC	MBC	MIC	MBC	MIC	MBC	MIC	MBC
*Listeria monocytogenes* ATCC 13932	>0.8	-	>0.8	-	>0.8	-	>0.8	-
*Bacillus cereus* ATCC 11778	>0.8	-	>0.8	-	>0.8	-	>0.8	-
*Streptococcus sanguinis*	>0.8	-	>0.8	-	>0.8	-	>0.8	-
*Streptococcus pyogenes*	>0.8	-	>0.8	-	>0.8	-	>0.8	-
*Streptococcus mutans*	>0.8	-	>0.8	-	>0.8	-	>0.8	-
*Pseudomonas aeruginosa* ATCC 10145	>0.8	-	>0.8	-	>0.8	-	>0.8	-
*Candida albicans* ATCC 10231	>0.8	-	>0.8	-	>0.8	-	>0.8	-

**Table 3:** OD values for bacterial strains *Pseudomonas aeruginosa* ATCC 10145 (1–2), *Listeria monocytogenes* ATCC 13932 (3–4), *Bacillus cereus* ATCC 11778 (5–6), and yeast *Candida albicans* ATCC 10231 (7–8) treated with _25_GQD^IPA^-EDA (top), _50_GQD^IPA^-EDA 50 (middle), and _200_GQD-1EDA (bottom).

	**1**	**2**	**3**	**4**	**5**	**6**	**7**	**8**	
A	0.94	0.97	0.24	0.23	0.56	0.58	0.53	0.52	600
B	0.91	0.96	0.21	0.22	0.52	0.53	0.52	0.57	600
C	0.88	0.87	0.23	0.22	0.5	0.52	0.7	0.63	600
D	0.77	0.7	0.23	0.23	0.5	0.51	0.7	0.63	600
E	0.68	0.7	0.23	0.22	0.49	0.48	0.62	0.57	600
F	0.8	0.72	0.25	0.2	0.5	0.51	0.55	0.59	600
G	0.83	0.75	0.17	0.2	0.5	0.49	0.58	0.59	600
H	0.05	0.38	0.05	0.06	0.05	0.05	0.06	0.11	600
	**3**	**4**	**7**	**8**	**9**	**10**	**11**	**12**	
A	0.83	0.85	0.3	0.32	0.46	0.5	0.42	0.43	600
B	0.87	0.93	0.21	0.27	0.45	0.45	0.51	0.49	600
C	0.9	0.9	0.18	0.25	0.48	0.49	0.57	0.59	600
D	0.88	0.76	0.13	0.17	0.4	0.46	0.48	0.47	600
E	0.69	0.69	0.14	0.17	0.53	0.52	0.53	0.49	600
F	0.9	0.63	0.15	0.15	0.47	0.47	0.49	0.5	600
G	0.69	0.58	0.13	0.41	0.42	0.45	0.59	0.58	600
H	0.05	0.05	0.05	0.05	0.05	0.05	0.05	0.05	600
	**3**	**4**	**7**	**8**	**9**	**10**	**11**	**12**	
A	0.97	0.94	0.37	0.41	0.54	0.56	0.46	0.38	600
B	0.9	0.86	0.31	0.32	0.54	0.55	0.43	0.37	600
C	0.82	0.77	0.25	0.26	0.57	0.52	0.52	0.5	600
D	0.68	0.63	0.22	0.19	0.48	0.47	0.49	0.49	600
E	0.63	0.59	0.17	0.18	0.45	0.45	0.47	0.53	600
F	0.59	0.6	0.16	0.19	0.45	0.47	0.53	0.55	600
G	0.61	0.63	0.15	0.16	0.44	0.47	0.6	0.48	600
H	0.05	0.05	0.05	0.05	0.05	0.05	0.06	0.05	600

## Data Availability

MDPI research data policies.
